# Gold Nanoparticles Dissolve Extracellularly in the Presence of Human Macrophages

**DOI:** 10.2147/IJN.S314643

**Published:** 2021-08-26

**Authors:** Sarah McCarrick, Klara Midander, Magdaléna Krausová, Ulrika Carlander, Hanna L Karlsson

**Affiliations:** 1 Institute of Environmental Medicine, Karolinska Institute, Stockholm, SE-171 77, Sweden

**Keywords:** dissolution, biosolubility, metal release, nanotoxicology, transformation, fate

## Abstract

**Introduction:**

Gold nanoparticles (AuNPs) have the potential to be used in various biomedical applications, partly due to the inertness and stability of gold. Upon intravenous injection, the NPs interact with the mononuclear phagocyte system, first with monocytes in the blood and then with macrophages in tissue. The NP–macrophage interaction will likely affect the stability of the AuNPs, but this is seldom analyzed. This study aimed to elucidate the role of macrophages in the biodissolution of AuNPs and underlying mechanisms.

**Methods:**

With an in vitro dissolution assay, we used inductively coupled plasma mass spectrometry to quantitatively compare the dissolution of 5 and 20 nm AuNPs coated with citrate or PEG in cell medium alone or in the presence of THP1-derived macrophages at 24 hours. In addition, we analyzed the cell dose, compared extra- and intracellular dissolution, and explored the possible role of reactive nitrogen species.

**Results:**

The results showed a higher cellular dose of the citrate-coated AuNPs, but dissolution was mainly evident for those sized 5 nm, irrespective of coating. The macrophages clearly assisted the dissolution, which was approximately fivefold higher in the presence of macrophages. The dissolution, however, appeared to take place mainly extracellularly. Acellular experiments demonstrated that peroxynitrite can initiate oxidation of gold, but a ligand is required to keep the gold ions in solution.

**Conclusion:**

This study suggests extracellular dissolution of AuNPs in the presence of macrophages, likely with the contribution of the release of reactive nitrogen species, and provides new insight into the fate of AuNPs in the body.

## Introduction

Gold nanoparticles (AuNPs) have unique properties that make them attractive in a broad spectrum of biomedical applications. The use of AuNPs in nanotherapeutics^1^,^2^ has been investigated for photothermal therapy,^1^ drug delivery,^2^ and imaging.^3^ Today, the most explored route of administration for these applications is intravenous injection. When AuNPs enter the circulation, they are distributed throughout the body.^4^,^5^ The majority of AuNPs will never reach their intended target organs. Instead, they are captured by cells of the mononuclear phagocyte system (MPS) including macrophages, especially in organs with fenestrated vasculature as in the liver and spleen.^6^ By altering AuNP properties, the distribution profile and cellular uptake can be modified. For example, prolonged circulation time can be achieved by coating with polyethylene glycol (PEG),^7^,^8^ and improved clearance is obtainable by decreasing the size to <6 nm.^3^,^9^ However, once trapped, AuNPs will reside within the MPS for extended periods with slow clearance.^6^,^9^,^10^ Even though a large fraction of the NPs in the blood end up in the MPS, little attention has been given to the fate and long-term effects of AuNPs in the MPS.

Stability is a crucial factor for nanotherapeutics to assure safe performance and fate. Gold is a suitable candidate, due to its inertness and low release of potentially toxic bioactive ions.^6^,^10^,^11^ Studies have demonstrated gold dissolution in the environment^12^,^13^ and in different biological systems for bulk gold^14^,^15^ and AuNPs.^16–18^ We have previously shown time- and size-dependent dissolution of citrate-coated AuNPs and that the presence of macrophages greatly enhanced the dissolution, particularly if they were triggered with lipopolysaccharide.^19^

Although some studies have demonstrated dissolution of AuNPs in biological settings, the underlying mechanisms are still unknown. Lysosomal degradation has been proposed as a primary mechanism of AuNP dissolution in both acellular systems^16^ and during long-term in vitro studies (up to 6 months).^18^ Larsen et al^14^ demonstrated the liberation of gold ions in membrane-like layers between metal and macrophages, pointing toward an extracellular dissolution process. Macrophages can recognize and engulf AuNPs by uptake mechanisms including endocytosis and phagocytosis.^6^ Furthermore, these cells have an oxidative metabolism and digest foreign invaders by producing reactive oxygen/nitrogen species (ROS/RNS), both in lysosomes and extracellularly. This includes the production of superoxide by the highly expressed enzymes NADPH oxidase and nitric oxide by nitric oxide synthase (iNOS), which together form peroxynitrite.^20–22^

This study aimed to explore the role of human-derived macrophages in the biodissolution of AuNPs, as well as underlying mechanisms. The specific aims were to 1) investigate size and coating dependence of AuNPdissolution in the presence of macrophages, 2) elucidate whether dissolution takes place mainly extra- or intracellularly, and 3) explore and hypothesize mechanisms involved in the macrophage-dependent dissolution of AuNPs.

## Methods

Particles were primarily characterized for size, morphology, and cellular uptake. This was followed by modification and performance validation of the in vitro dissolution assay to answer the research questions of aim 1 and 2. Finally, a mechanistic investigation using acellular systems was performed in relation to aim 3.

### Chemicals

RPMI 1640 (R0833), phorbol 12-myristate 13-acetate (PMA; P1585), SIN1 hydrochloride (Calbiochem 567028), sodium chloride (NaCl, 31434M) and proteinase K (P2308) were purchased from Sigma-Aldrich. FBS (10270-106), penicillin–streptomycin (15140−122), L-glutamine (25030081), PBS (10010−015), and trypsin (15400-054) were purchased from Thermo Fisher Scientific. Stock solutions of gold ions (HAuCl_4_, 1,000±6 μg Au/mL in 2% HNO_3_ [v:v]), and bismuth (1,004±6 μg/mL in 5% HNO_3_) were obtained from Spectrascan Teknolab. Nitric acid (67% HNO_3_, Normatom for trace-metal analysis) was from VWR International and hydrochloric acid (37% HCl, ACS for analysis) from Merck. Ultrapure water used in the study was produced using PureLab Flex 3 from ELGA LabWater (18.2 MΩ/cm).

### AuNPs

Four types of BioPure AuNPs were purchased from NanoComposix. The particles were spherical with a diameter of 5 or 20 nm and coated with either PEG or citrate. They were delivered in the form of a stock solution of 1 mg/mL, with ultrapure water as the solvent. According to material specifications from the supplier, particles were monodispersed and unagglomerated, with a purity of 99.9%. The pH range for AuNP stock solutions was 5.6–6.6. The ζ-potential was −24 mV for 20 nm PEG-coated AuNPs and −46 mV for 20 nm citrate-coated AuNPs. Information on size distribution by DLS and TEM was provided by the manufacturer (in the case of 5 nm AuNPs, no results of ζ-potential or DLS were reported). To ensure that no gold ions were present in the AuNP stock solution, 5 µL was withdrawn, added to 995 µL cell media and centrifuged (10,000 rpm/9,600 *g* for 10 minutes) using a spinfilter. The concentration of gold in the particle-free supernatant was below the limit of detection (LOD; n=2) for all particle types, as analyzed using ICP-MS. To exclude the interference of bacterial endotoxin contamination, a Pierce Chromogenic Endotoxin Quant Kit (Thermo Fisher Scientific) was used according to manufacturer instructions. The results demonstrated endotoxin levels to be ≤2.5 EU/mL for all particles tested. This is in line with the NanoComposix guarantee of <5 EU/mL for particle sizes 20 nm and less.

### Cell Culture and Exposure

THP1 is an immortalized human leukemia monocyte cell line that originated from a 1-year-old male infant, and is one of the most established cell lines for investigations regarding monocytes and macrophages in the cardiovascular system.^24^ Upon stimulation of the THP1 monocytes with PMA, they differentiate into a macrophage phenotype (THP1*) with a low level of proliferation, which can be of value for studying different timepoints. The cells were obtained from the American Type Culture Collection and maintained in RPMI medium supplemented with 10% FBS, 1% penicillin–streptomycin, and 1% L-glutamine. The cells were cultured in a standing T75 cm^2^ flask (VWR 734-2313) in a standard humidified incubator at 5% CO_2_ and 37°C and subcultured every 2–3 days. Prior to experiments, the desired density of 1,000,000 cells/well in a six-well plate (Corning CLS3506), or 500,000 cells/well in a 24-well plate (Corning CLS3527) of THP1 monocytes were incubated with 5 ng/mL PMA for 24 hours to promote differentiation into adhesive THP1 macrophages, ie THP1* cells. PE-tagged mouse antihuman CD11b/Mac1 (BD Pharmingen) was used as a marker to confirm macrophage differentiation with PMA by flow cytometry (BD Accuri C6 Plus Flow Cytometer). The surface marker CD11b was found to be expressed to a greater extent following PMA differentiation than undifferentiated THP1 cells (Figure S1).

For exposure, following differentiation into THP1* cells in the 24-well plate, the PMA-containing medium was replaced with 1 mL fresh medium, and 5 µL AuNP stock solution (1 mg/mL) was added directly to each well, resulting in a total dose of 5 µg AuNPs/mL (approximately 2.5 µg AuNPs/cm^2^). For visualization of cellular dose (six-well plate), the PMA -containing medium was replaced with 2 mL fresh medium, and 23.8 µL AuNP stock solution was added to reach the equivalent of 2.5 µg AuNPs/cm^2^. The dose (5 μg/mL) and time point (24 hours) used in this study did not affect the viability of the THP1* cells (Figure S2).

### TEM Analysis of AuNPs and Cells Exposed to AuNPs

The primary size and shape of AuNPs were studied using TEM (Hitachi HT7700) operating at 100 kV. Samples for imaging were prepared from a particle suspension of 10 µg/mL in deionized water, and applied to 200-mesh TEM copper grids with formvar/carbon support films (Ted Pella) following drying in ambient laboratory conditions.

Uptake of AuNPs in THP1* cells was studied using TEM (FEI Tecnai 12 Spirit BioTwin) at 100 kV. Cell samples were prepared from THP1* in a six-well plate. After 24 hours of exposure, the medium was withdrawn and wells washed with PBS twice before addition of trypsin, followed by 3 minutes incubation to detach the cells. Fresh medium (1 mL) was added to the wells, and all content was subsequently collected in 1.5 mL microtubes (Eppendorf safe-lock tubes) and centrifuged for 4 minutes at 2,000 rpm (400 *g*). The supernatant was subsequently removed and each pellet fixed with 1 mL 2% osmium tetroxide. Sections of the cell pellets were cut (60–80 nm) and contrasted with uranyl acetate and lead citrate prior to TEM investigation.

### Nanoparticle-Tracking Analysis of AuNPs

To obtain the hydrodynamic size distribution of AuNPs in the cell medium, a NanoSight LM10 platform with an sCMOS camera equipped with a 405 nm laser was used for NP-tracking analysis (NTA). The control measurement for the instrument using 23 nm latex NPs gave reliable results, and measurement of ultrapure water revealed little particle contamination. This analytic technique uses properties of both light scattering and Brownian motion, and the 2.3 analytical software package tracks many particles individually and uses the Stokes–Einstein equation to calculate their hydrodynamic diameters. However, this possibility is limited to certain conditions, ie, a particle-concentration range of 10^8^ to 5×10^9^/mL, which in our case required substantial dilution (1:20 in ultrapure water) of the particle–medium solutions used for experiments. Triplicate measurements were performed for each of the four AuNPs at camera level 13 with 60-second capture at constant flow (injection speed of syringe pump set to 50) and detection threshold 3 at room temperature (22.3°C). The viscosity of water + 5% medium was given as the input value to obtain results of hydrodynamic size distribution of AuNPs in medium.

### In Vitro Dissolution Assay

**Figure 1:**
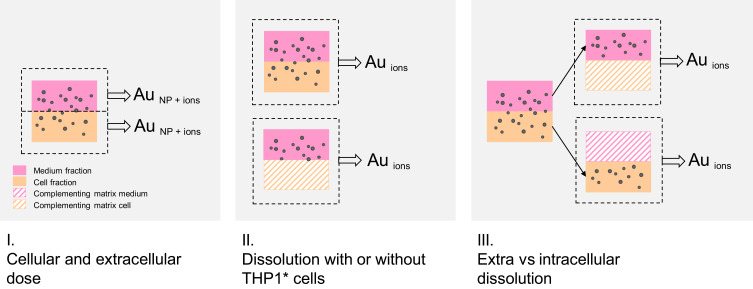
The experimental approach comprised three stages, in which total Au or dissolved AuNPs were assessed in different fractions following 24 hours’ incubation of AuNPs. In part I, we measured total Au (NPs and ions) in intra- (cellular dose) and extracellular fractions separately. In part II, the dissolution of AuNPs, ie, the release of gold ions, with or without the presence of THP1 macrophages was quantified. In part III, we measured the dissolved AuNPs in the presence of macrophages in extra- and intracellular fractions separately. These three experimental parts were followed by sample preparation and metal analysis using ICP-MS.

With the aim of quantifying AuNP dissolution in the presence of macrophages and to determine whether dissolution occurred intra- or extracellularly, we performed a series of experiments (Figure 1). The key challenge of our experimental approach was to separate the dissolved gold ions from AuNPs in the different fractions of the in vitro experiments. The separation method (centrifugation only) used for RAW264.7 cells by Carlander et al^19^ was found not to be stable enough using THP1*, particularly for the PEG-coated AuNPs. The problematic separation of dissolved gold from the particle–cell matrix was overcome by using a 10 kDa spin filter (corresponding to pore diameter of 1.42 nm)^25^,^26^ during centrifugation. Our previous attempts to filter the samples was shown to remove all gold ions in control experiments.^19^ However, inspired by Koltermann-Jülly et al,^27^ we added a step of protein digestion (proteinase K) following lysis, which enabled the use of a 10 kDa spin filter to enable the separation of particles and dissolved ions. The modified separation method was adopted in part II and III, and is described in detail in the following sections (Figure 1).

#### Cellular and Extracellular Doses of AuNPs (Part I)

To determine the cellular dose of AuNPs in THP1* cells following 24 hours of exposure, medium and cells were collected and treated separately. The cell fraction was treated with 1 mL concentrated aqua regia (25% HCl, 75% HNO_3_) in the well for 1 hour. This was transferred to a 2 mL safe-lock tube, and an additional 0.5 mL concentrated aqua regia used for washing the well was transferred to the same tube and digested for at least 24 hours prior to analysis. Immediately prior to metal analysis, 100–500 µL of the aqua regia–treated cell fraction, ie, the cellular dose, was diluted with 4.5–4.9 mL ultrapure water in 15 mL Falcon tubes (Corning CLS430791). For the extracellular (remaining) dose (ie, samples of solely the medium), 0.25 mL of the sample was treated with 1 mL aqua regia for at least 24 hours. A volume of 0.5 mL of the treated samples was diluted with 4.5 mL ultrapure water prior to metal analysis with ICP-MS.

#### Dissolution with and without Macrophages (Part II)

To enable comparison of AuNP dissolution in the presence and absence of macrophages, AuNPs were incubated with THP1* cells or solely medium in 24-well plates for 24 hours. For samples without cells, the AuNPs and released ions in medium were transferred to a microtube together with a solution of lysed cells (prepared from unexposed cells) to complement the matrix, as described in Carlander et al.^19^ All samples were further treated according to the protocol described in the method section Separation of Dissolved Gold from AuNPs in Cellular Matrix.

#### Extra vs Intracellular Dissolution (Part III)

To explore AuNP dissolution occurring intracellularly vs extracellularly, AuNPs were incubated with THP1* cells for 24 hours. Cells and media were collected separately for detection of intra- and extracellular dissolution, respectively. To complement the sample matrix, fresh medium (1 mL) was added to the cell fraction and a solution of cell lysis (from unexposed cells) added to the medium fraction. The samples were further treated according to the protocol for separation described in the next method section on Separation of Dissolved Gold from AuNPs in Cellular Matrix.

#### Separation of Dissolved Gold from AuNPs in Cellular Matrix

**Figure 2:**
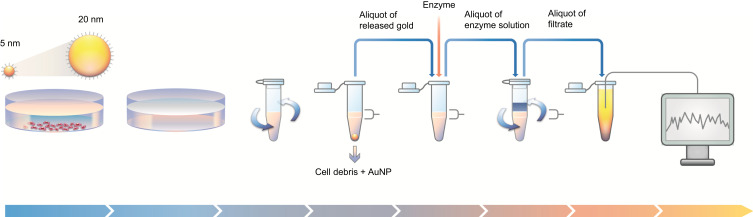
Schematic overview of the experimental procedure, illustrating the steps used for detecting gold dissolution in the macrophage cell system. Initially, AuNPs (5 or 20 nm, with citrate or PEG coating) were incubated with differentiated THP1 macrophages for 24 hours. Next, the cells were lysed prior to centrifugation (13,000 rpm, 30 minutes) of the medium–lysate–AuNP mix. The upper part of the supernatant was then collected, incubated with proteinase K (0.1 mg/mL) for 45 minutes, and spun through a 10 kDa spin filter (10,000 rpm, 10 minutes). The filtrate was then collected, treated with aqua regia, and analyzed for gold content using ICP-MS.

Following exposure; the cell medium from each well was collected in individual 1.5 mL safe-lock microtubes. Ultrapure water (200 µL) was added to the wells, cells were scraped off using a pipette tip, and then mechanically lysed by five passages through a 27 G needle (Microlance 3, 27G3/4, 0.4×19 20 302200, Becton Dickinson) before being added to the same tube as the medium. The wells were rinsed with an additional 200 µL ultrapure water and added to the tube. To separate dissolved gold from the remaining NPs and cell debris, the mixtures were centrifuged at 13,000 rpm (16,060 *g*) for 30 minutes at 4°C, after which the upper part of the supernatant was collected into a new microtube and incubated with proteinase K (0.1 mg/mL) for 45 minutes at 37°C. Finally, the supernatant was spun through a 10 kDa centrifugation filter (VWR 82031-348) at 10,000 rpm (9,600 *g*) for 10 minutes, and 0.25 mL of the filtrate was withdrawn and prepared for metal analysis by ICP-MS, see Preparation for Metal Analysis of Part II and III. The experimental procedure for separation of dissolved gold from AuNPs in cellular matrix is presented in Figure 2.

To initially explore the NP-separation efficiency of our method, samples of AuNPs and a solution of lysed cells (prepared from unexposed cells) were at 0 hours treated according to the protocol for separation described (Figure 2) and gold was analyzed in the filtrate. This experiment was done to confirm that the filtrate we use for measurement did not contain any NPs, only ions. The results showed separation efficiency to be high, since gold levels in all digested filtrate samples were below the LOD, except for two of the six samples for 5 nm citrate-coated AuNPs: 0.16 and 0.22 wt% of added dose.

Also, to confirm that the method allowed detection of released ions, THP1* cells were exposed to 0.05, 0.25, or 1 µg gold ions (in the form of HAuCl_4_) for 24 hours and harvested according to the protocol described in this section and in Figure 2. Ion recovery was calculated as detected gold in filtrate relative to added gold. We detected approximately 20 wt% (17.8–22.7 wt%) of the added gold ions in the filtrate, independently of the dose added (see Table S1 and Figure S3). This suggests that the use of the 10 kDa filter can be employed together with protein digestion to provide a stable separation method, although dissolved gold will be largely underestimated.

#### Preparation for Metal Analysis of Parts II and III

Sample types from part II and III experiments contained a mixture of medium and lysed cells and were prepared for metal analysis with ICP-MS. A volume of 0.25 mL from the samples was digested with 1 mL aqua regia in a 2 mL safe-lock microtube overnight at room temperature in a ventilated hood. The next day, prior to analysis, 0.5 mL was transferred to a 15 mL tube (Corning CLS430791) and diluted with 4.5 mL ultrapure water.

### Acellular Dissolution Assay to Test the Role of Peroxynitrite

AuNPs (5 µL from stock solution) were incubated in 1 mL cell medium, PBS or NaCl (9mg/mL) with or without peroxynitrite. Peroxynitrite was generated by addition of peroxynitrite donor SIN1 in solution, producing both nitric oxide and superoxide upon decomposition in aqueous solutions. Aliquots of fresh SIN1 solution (6.2 μL stock, 10 mg/mL in DMSO, resulting in a final concentration of 300 µM) was initially added six times to the samples every 1.5 hours, followed by vortex mixing. After a total of 24 hours’ incubation at 37°C, samples were transferred to a 10 kDa spin filter and centrifuged at 10,000 rpm (9,600 *g*) for 10 minutes. The filtrate (0.25 mL) was collected and treated with 1 mL aqua regia overnight. Prior to metal analysis, 0.5 mL was further diluted with 4.5 mL ultrapure water.

### Gold Analysis with ICP-MS

Standard solutions of 0, 0.1, 0.5, 1, 5, 10, 50, and 100 μg Au/L were prepared from a gold stock solution by dilution with 2% aqua regia. Bismuth was added as an internal standard at a concentration of 5 μg/L, thereby enabling the correction of measured gold concentrations based on bismuth recovery in samples.

ICP-MS (Thermo Fisher Scientific iCAP Q) was used to measure gold concentrations in the samples. Levels of ^197^Au and ^209^Bi isotopes were quantified as the average of triplicate readings in each sample in KED mode using argon as vector gas and helium as collision gas. For further details, see Carlander et al.^19^ The LOD was evaluated for each sample (diluted aqua regia–digested cell and medium samples, PBS and NaCl matrices, and ultrapure water) as three standard deviations of blank matrix samples, and was found to be 0.013–0.055 µg Au/L. The limit of quantification (LOQ) referred to ten standard deviations of blanks. Recovery of the internal standard was typically 70%–90%, due to matrix effects.

### Statistical Analysis

All results are presented as means ± SD. Results below the LOD were assigned the exact value of the LOD in further calculations, while those below the LOQ were used without modification. Statistical differences between different groups were evaluated in GraphPad Prism 8.0.1 (244) using nonparametric Mann–Whitney tests (unpaired, two-tailed). Differences were considered significant at p<0.05.

## Results and Discussion

### TEM Imaging Confirms Sizes of 5 and 20 nm

**Figure 3:**
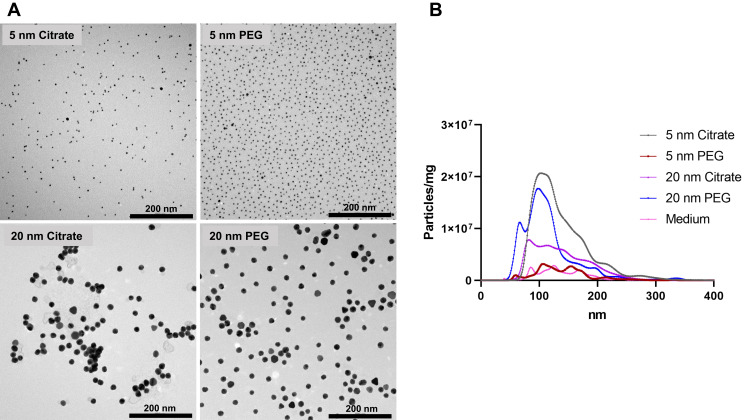
(**A**) TEM of AuNPs of varying size (5 or 20 nm) and coating (citrate or PEG). (**B**) Distributions of hydrodynamic particle diameter of the AuNPs in cell medium by NTA.

TEM verified the primary particle size of the AuNPs provided by the manufacturer through visual evaluation (Figure 3A). Evaluating the degree of particle agglomeration using TEM is precarious, since agglomeration can take place during drying of the sample. NTA can provide complementary information on hydrodynamic size distribution of particles, including agglomeration.^28^ NTA has been reported to record sizes as small as 20 nm,^29^ and was here used to study agglomerates of the AuNPs in 5 nm and 20 nm (ie, all NPs >20 nm), in diluted cell media. Results were obtained for citrate-coated AuNPs (5 and 20 nm) and 20 nm PEGylated AuNPs, indicating agglomerates of around 100 nm in all cases; however, a less distinct peak was observed for 20 nm citrate. The 5 nm PEGAuNPs were not detectable using NTA, which indicated that particles were unagglomerated. It should be stressed that the test conditions for NTA were different from those in our experiments.

### Citrate-Coated AuNPs Are Taken Up to a Higher Extent in Macrophages Than PEG-Coated AuNPs

**Figure 4:**
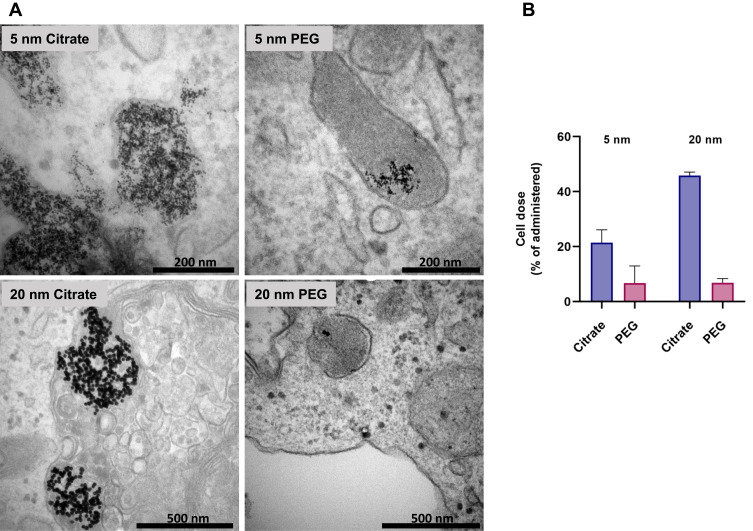
(**A**) TEM of AuNPs in macrophages, taken following 24 hours’ exposure of THP1 macrophages to 5 μg/mL AuNPs. (**B**) Cellular dose of Au depending on particle size and coating (% of administered). The bars (n=6) show the cellular dose of Au in the form of AuNPs or dissolved ions in the cells, or attached to cell surfaces after 24 hours’ exposure.

To investigate uptake and intracellular localization of AuNPs and to characterize NPs intracellularly, TEM was performed on macrophages exposed to AuNPs (Figure 4A). The results showed that all AuNPs were taken up and localized mainly in membrane-like endosomal structures, which is in line with what has been observed following citrate-coated AuNP exposure to RAW macrophages.^19^ The citrate-coated AuNPs (5 and 20 nm) were found to be highly agglomerated, and endosomes typically contained hundreds of agglomerated AuNPs. The 5 nm PEG AuNPs were found in lower numbers than the citrate-coated ones, and very few (one or two) of the 20 nm PEG AuNPs were observed in the endosomal structures.

To confirm the TEM images in a quantitative manner, total gold (AuNPs and dissolved gold ions) of the cellular (ie, in the cells or attached to the cell surface) and extracellular doses were measured separately using ICP-MS (part I, Figure 1). In line with the TEM images, the cellular dose was higher for citrate-coated AuNPs than the PEG-coated ones (Figure 4B and Table S2). Furthermore, for citrate-coated AuNPs, the cellular dose was higher for larger particles (20 nm, 46 wt% of added dose of AuNPs) than smaller particles (5 nm, 22 wt% added), while the PEG-coated particles showed a similar cellular dose of approximately 7 wt% for both 5 and 20 nm (Figure 4B). The collective recovery of total gold in cellular and extracellular doses was found to be >100 wt% (see Figure S4). It should be noted that cellular doses measured according to this procedure are likely to reflect both uptake into cells and sedimentation on the cells.

Previous studies have shown that the uptake of AuNPs in cells is strongly dependent on size. For example, Chithrani et al^30^ showed that with variously sized transferrin-coated AuNPs, 50 nm AuNPs were taken up at the highest rate and extent compared to both smaller and larger AuNPs in various mammalian cells. For citrate-coated AuNPs, the trend is that larger particles are internalized to a higher extent than smaller one,^19^,^31^ which is consistent with our results. Unlike citrate-coated AuNPs, we observed no clear size-dependency for uptake of PEG AuNPs. This is different from other studies, where size has been demonstrated to be important for cellular uptake of variously sized PEG AuNPs <100 nm, where uptake has been shown to increase with size in RAW 264.7^32^, ^33^ and J774A.1 macrophages.^7^ Differences in surface chemistry, cell lines, and experimental conditions among studies may to some extent account for this.

Protein adsorption occurs at the NP surface upon blood and tissue contact, which influences uptake efficiency and mechanisms in macrophages.^6^ Surface modifications, such as PEGylation, are used to decrease unspecific protein adsorption and prolong circulation. In a study comparing protein coronas of AuNPs with different surface modifications, AuNPs with PEG modification showed no protein adsorption, while citrate coating resulted in a 6–8 nm thick corona.^8^ In agreement with our results, PEGylated NPs have been shown to have decreased uptake compared to unmodified AuNPs in macrophages^34^ and A549 cells^35^. In a study by Walkey et al,^7^ increased PEG density in the coating of AuNPs was found to decrease total serum adsorption, and together with size determined mechanisms and efficiency of macrophage uptake. This should be further explored in future studies, as it can impact dissolution of AuNPs.

### Macrophages Increase Dissolution of AuNPs Dependent on AuNP Size, but Not Coating

**Figure 5:**
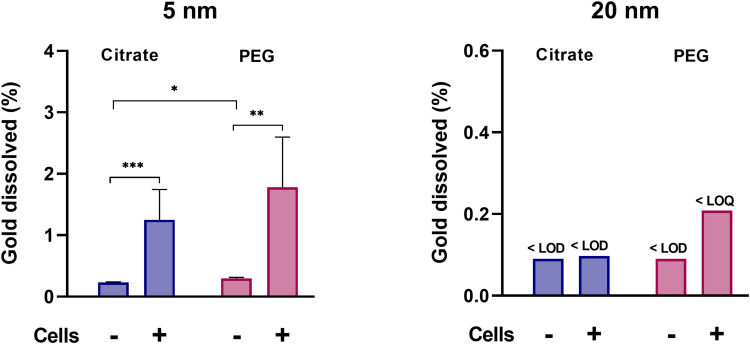
Dissolution of 5 and 20 nm AuNPs with citrate or PEG coating with or without the presence of macrophages (in vitro dissolution assay, part II). The AuNPs were incubated for 24 hours in cell medium alone (n=6) or with differentiated THP1 macrophages (n=12). *p<0.05; **p<0.01; ***p<0.001).

Results from investigating the influence of particle size, coating, and the role of macrophages in the dissolution of AuNPs (part II, Figure 1) showed comparable levels of dissolved gold for the 5 nm citrate- and PEGAuNPs (Figure 5 and Table S3). The presence of THP1* cells resulted in approximately a fivefold increase (*p*<0.001) in dissolved gold compared to levels in cell medium alone: from 0.23 to 1.25 wt% of the added AuNPs for citrate-coated AuNPs and from 0.3 to 1.78 wt% for PEGAuNPs. The increase due to the presence of macrophages was not as pronounced for the 20 nm AuNPs during the 24-hour experiment: no dissolution of 20 nm PEG AuNPs was detected in cell medium only (<LOD), but low levels (0.21 wt% of added, <LOQ) was detected in the presence of macrophages. For 20 nm citrate-coated AuNPs, practically no dissolution was detected, with levels below the LOD in cell medium alone and in the presence of macrophages.

These findings are in line with our previous results on macrophage-assisted dissolution of AuNPs,^19^ where 24 hours’ incubation of 5 nm citrate-coated AuNPs with RAW macrophages resulted in 1.6 wt% dissolved gold, similar to the 1.25wt% in the present study. However, it should be stressed that experimental conditions, including AuNP batch, separation method, cell lines, cell medium, cell uptake, and ion recovery, differed between the studies. In a study on rats by Lopez-Chaves et al,^17^ smaller AuNPs than initially administered were detected in cells and lipid droplets. In combination with low-molecular-weight Au species detected in organs, this suggests degradation through biological or chemical mechanisms, and was further proposed to be due to the phagocytic activity of macrophages in spleen or Kupffer cells. The importance of macrophages in dissolution/degradation has been demonstrated in a few studies for other NPs, including MnO_2_ NPs,^36^ carbon nanotubes,^37^ SrCO_3_, and BaSO_4_.^27^ It can be hypothesized that the higher dissolution of 5 nm than 20 nm AuNPs, especially in the presence of macrophages, is likely to be related to increased surface area and reactivity.^38^ If ROS and ligands are present, oxidation of these surface gold atoms can take place. As such, thermodynamically, 5 nm particles are more soluble than 20 nm ones.

To our surprise, we found that PEG AuNPs had similar dissolution as citrate-coated AuNPs. Our findings are somewhat remarkable, taking into consideration that the cellular dose was lower in 5 nm PEG AuNPs than citrate-coated AuNPs. Due to the higher agglomeration observed for citrate-coated AuNPs, one can expect higher sedimentation on the cells. Together with the higher uptake observed for citrate-coated AuNPs, this should theoretically result in more extensive contact with the cells and thus higher cell-assisted dissolution compared to PEG AuNPs, with the steric barrier limiting agglomeration and sedimentation. Depending on surface species and mode of attachment, surface coatings may modify dissolution of NPs, and capping agents have further been shown to considerably impact dissolution.^39–41^

Taken together, our results demonstrated a size-dependent dissolution of AuNPs in the presence of macrophages, where smaller AuNPs dissolved to a greater extent compared to larger ones, irrespective of surface coating. This size-dependent effect could not be explained by the differences in surface area (Table S3).

Like our previous study, we here focused on the 24-hour time point, while in vivo AuNPs can reside inside macrophages over extended periods. Therefore, we encourage further observations that explore long-term dissolution behavior of AuNPs, both in vitro and in vivo.

### Dissolution of AuNPs Occurs Mainly Extracellularly

**Figure 6:**
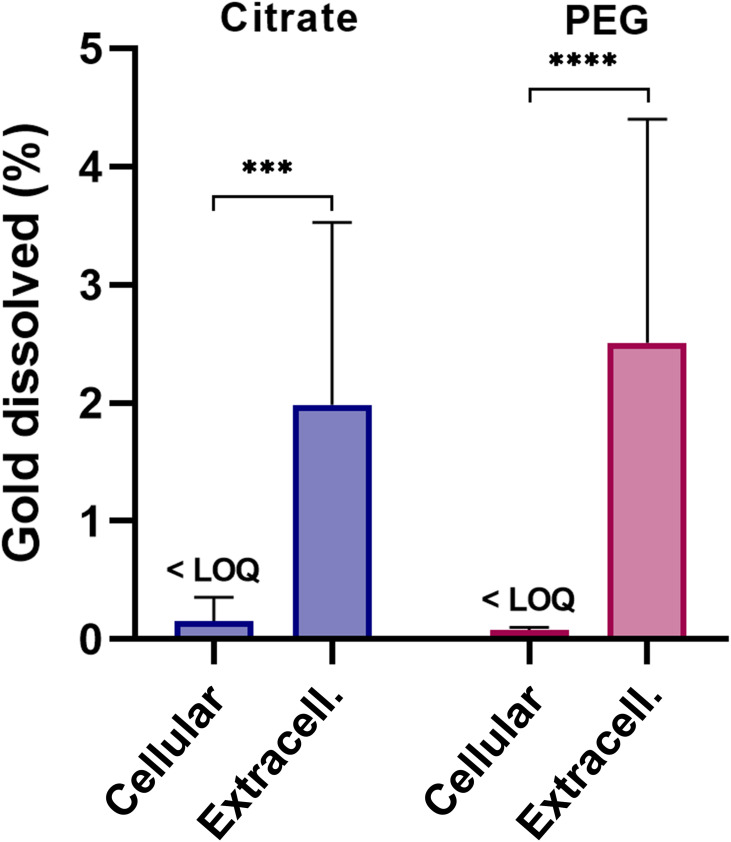
Cellular versus extracellular dissolution of 5 nm AuNPs with citrate or PEG coating (part III). The AuNPs were incubated for 24 hours in cell medium with differentiated THP1 macrophages for 24 hours, and cells and media were collected and analyzed for gold ions separately (n=9). *p*;*p*; ***p<0.001; ****p<0.0001.

The fact that coating — independently of the level of uptake observed — had no effect on macrophage-assisted dissolution led to the hypothesis that the dissolution may not be dependent on uptake. Therefore, we next investigated whether the dissolution process of 5 nm AuNPs occurs extra- or intracellularly (part III, Figure 1). In these experiments, the medium and cell fractions of exposed cells were analyzed for dissolved gold separately, thus representing extracellular and intracellular dissolution, respectively. The results demonstrated that dissolved gold was found to a significantly higher extent in the extracellular fraction, ie, in the medium (1.98 and 2.51 wt%) than the intracellular fraction for 5 nm citrate-coated (<LOQ) and PEG (<LOQ) AuNPs, respectively (Figure 6 and Table S4). It should be noted that we controlled for possible differences in matrix during separation by adding cell lysate to complement the medium fraction.

The cellular dissolution of AuNPs has been proposed to occur intracellularly in lysosomes. Sabella et al^16^ proposed the concept of a lysosomal-enhanced Trojan horse effect to be the mechanism behind gold dissolution. Their study demonstrated time-dependent acellular release of gold ions ion in acidic conditions (pH 4.5), but not at neutral pH. The cytotoxic effects of AuNPs in HeLa cells were further found to be reduced by coexposure to lysosomotropic agents, indicating lysosome-dependent toxicity. Balfourier et al^18^ recently demonstrated long-term lysosomal degradation of AuNPs up to 6 months in primary fibroblasts, followed by a gold-recrystallization process generating self-assembled nanoleaves. The degradation was proposed to be mediated by NADPH oxidase, producing ROS in lysosomes. A major difference with our study compared to Sabella et al^16^ and Balfourier et al^18^ is that we quantitatively measured dissolution inside and outside cells. Sabella et al^16^ focused on quantitative measurements of lysosomal dissolution in an acellular system and Balfourier et al^18^ on qualitative visualization of dissolution inside fibroblasts with the help of TEM.

Macrophages elicit oxidative burst, where ROS/RNS are released both in lysosomes and extracellularly.^20–22^ In line with our results on extracellular dissolution, it has been demonstrated that the particle–cell interface of bacteria increases local concentrations of metal ions released from NPs, emphasizing that new quantitative techniques should be developed to determine additional dissolution of NPs due to particle–cell interaction.^42^ In a study by Larsen et al,^14^ macrophages were grown on slabs or grids of 24-carat gold, and were found to liberate gold ions in a membrane-like layer in the interface between the metal and the macrophages. They suggested that hypochlorite, formed during oxidative burst, oxidizes and dissolves gold, and in the presence of thiocyanate aurocyanide is formed.

From this, we can conclude that the short-term (24 hours) macrophage-assisted dissolution of AuNPs observed in this study took place mainly extracellularly. To our knowledge, this is the first time extra- vs intracellular dissolution has been characterized and quantified for AuNPs in the presence of macrophages. Knowledge of the location for initial dissolution of AuNPs is important for better understanding of the fate of AuNPs.

### Peroxynitrite Generator SIN1 Enhances Dissolution of AuNPs in Medium and PBS, but Not in NaCl

Based on our results of macrophage-assissted dissolution of 5 nm AuNPs occuring extracellularly, we were curious about any possible mechanisms involved in AuNP degradation, including the ability of macrophages to elicit oxidative burst. We have previously showed the ability of an acellular hydroxylradical–generating system to enable Au dissolution when the reaction took place in a liquid with ligands.^19^ Findings by Kagan et al^37^ demonstrating peroxynitrite-mediated degradation of single-walled carbon nanotubes inspired us to test the ability of peroxynitrite-generating systems to biodegrade AuNPs. One of several oxidative agents, peroxynitrite (redox potential 1.4)^43^ is generated via a reaction between superoxide radicals and nitric oxide, and can thus be produced in high quantities by activated macrophages. PMA-differentiated THP1* cells have been shown to produce nitric oxide or species thereof without lipopolysaccharide stimulation.^24^,^37^,^44^ Therefore, to test whether peroxynitrite in principle could enable the dissolution of AuNPs, we simultaneously produced superoxide and nitric oxide by the decomposition of SIN1 in cell media, PBS, and NaCl (9 mg/mL). Due to the low levels of dissolution observed for 20 nm AuNPs with or without the presence of macrophages, we investigated only 5 nm AuNPs in these experiments.

**Figure 7:**
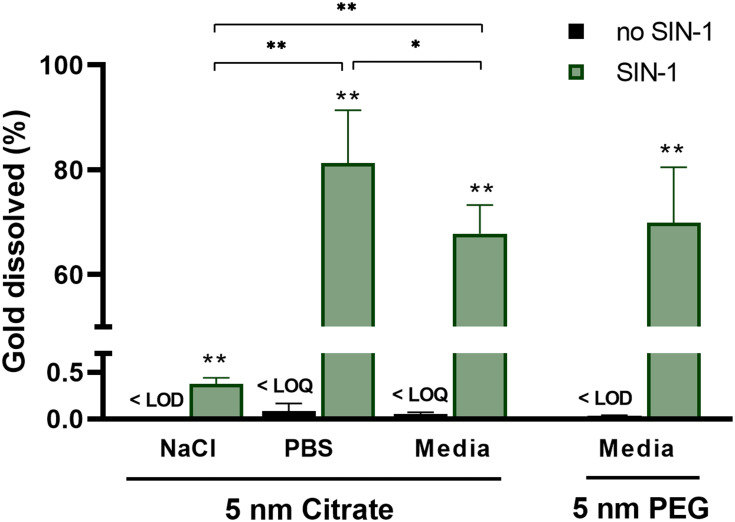
Dissolution of 5 nm citrate or PEG AuNPs in a simplified test system, ie, in the absence of macrophages. AuNPs were incubated with or without peroxynitrite generator SIN1 in medium, PBS, or NaCl (9 mg/mL) for 24 hours (n=6). *p<0.05, **p<0.01 Asterisks indicate significance versus the same solution without SIN1 unless otherwise indicated. SIN1, at a concentration of 300 µM, was added six times every 1.5 hours for the first 7.5 hours of the total incubation of 24 hours.

We found that dissolution of 5 nm AuNPs coated with PEG or citrate was significantly higher in all solutions where peroxynitrite was generated (Figure 7 and Table S5). Peroxynitrite or species thereof apparently dissolved small AuNPs, even though redox potential for bulk gold (1.69) is higher than that for peroxynitrite (1.4). However, by reducing size and adding appropriate ligands, the surface redox potential of AuNPs can be decreased to <1.4,^45–49^ which can explain why peroxynitrite can oxidize gold.

When citrate-coated AuNPs were incubated with peroxynitrite in PBS or medium, the dissolution increased approximately 200-fold compared to that from incubation with peroxynitrite in NaCl solution. Higher dissolution was also seen in PBS (80 wt%) than medium (70 wt%). Further, we observed no difference between dissolution for PEG AuNPs and citrate-coated AuNPs in cell medium, which cohered with our in vitro results for AuNPs in the presence of macrophages. Once dissolved, gold ions easily reduce back to elementary gold, unless they are stabilized by ligands. Based on our results, PBS and cell medium have the ability to stabilize gold ions in solution. Exactly how this occurs needs to be further explored in future studies. Based on our observations, it is possible that peroxynitrite in PBS may be used to simulate biostability of AuNPs in biological systems.

### Implications and Perspectives

**Figure 8:**
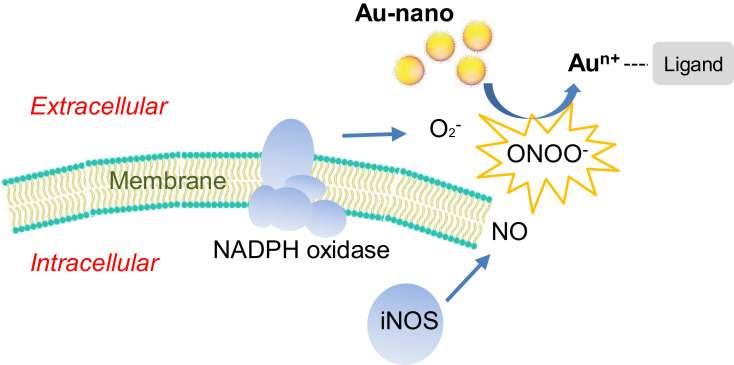
Proposed mechanism of extracellular AuNP dissolution in the presence of macrophages. NADPH oxidase and iNOS of activated macrophages produce and release superoxide (O_2_^–^) and nitric oxide (NO), respectively, that together form peroxynitrite during the process of oxidative burst. Peroxynitrite further dissolve small AuNPs.

The aim of this study was to investigate the influence of human-derived macrophages in the biodissolution of AuNPs and explore underlying mechanisms. Our results suggest that during a short period (24 hours), AuNPs dissolved in the presence of macrophages and dissolution took place mainly extracellularly. We have also shown that peroxynitrite, generated during oxidative burst, can initiate oxidation of AuNPs, but a ligand is required to prevent dissolved ions from reducing back to elementary gold. This generates a new proposed mechanism behind gold dissolution, apart from the lysosomal degradation suggested by Sabella et al^16^ and Balfourier et al.^18^ The proposed mechanism is summarized in Figure 8.

This study used an in vitro assay to quantitatively assess NP dissolution in the presence of human-derived cells. This assay afforded us the opportunity to separate intra- and extracellular fractions. To the best of our knowledge, no previous studies have presented an experimental approach generating quantitative results for dissolved gold following incubation of AuNPs with human macrophages, particularly enabling the separation of intra- and extracellular dissolution. The possibility of measuring cellular dissolution is the main strength of our assay, whereas most other studies investigating cellular dissolution have mainly used various imaging techniques and thus presented data in a more qualitative manner.

In accordance with Carlander et al,^19^ a major challenge in this study was the separation of the dissolved gold from the remaining AuNPs in the complex matrix of our in vitro model. With the introduction of the additional steps of protein degradation and ultrafiltration, separation was enabled. However, ion recovery was only about 20 wt%, and thus the results are considered a large underestimation (theoretically, values can be up to fivefold that). Ion recovery was lower than in our previous study, where it was around 60 wt% after 24 hours. This difference is probably a result of the new separation method, where the complex cellular matrices of our experiments are assumed to cause the low recovery and ions bound to proteins >10 kDa will be captured by the filter. By altering experimental parameters, such as incubation time, concentration of proteinase K, and filter size, ion recovery may be improved in future.

The relatively low uptake of AuNPs in THP1* cells observed in combination with the low recovery of our separation method may mean our method is not sufficient for detecting cellular dissolution, at least not in the short period studied here. The same may be true for the dissolution of 20 nm AuNPs, where lower dissolution was generally observed. Due to the levels measured being too close to the LOD for 20 nm AuNPs, it was impossible to explore intra- vs extracellular dissolution further. However, this can possibly be investigated in future studies by using longer exposure times and optimization to increase recovery of the separation method.

AuNPs are widely used and investigated for biomedical applications; however the fate of AuNPs in humans remains unclear and requires further attention. To optimize and design the target and function of applied AuNPs, the fate and biodissolution are important parameters. In conflict with the expected stability and inertness of AuNPs, our study demonstrates that biodissolution of small (5 nm) AuNPs occurs even within 24 hours. Dissolution may change properties and decrease size of the administered AuNPs, which may alter the intended function, as well as their fate and toxicity in the body. AuNPs have been demonstrated to be toxic, and particularly ultrasmall AuNPs have been shown to be of concern regarding toxicity.^50^

When AuNPs are dissolved, gold ions are released. Free gold ions are unstable, and thus if released in vivo, they will interact with the biological environment, eg, in reduction back to a nonionic form of gold or binding to a ligand.^45^,^49^ This could potentially interfere with and alter biological or cellular processes, which may lead to anti-inflammatory/immunosuppressive effects.^50–52^ The fact that we demonstrated short-term dissolution of AuNPs to occur mainly extracellularly is novel, and requires further investigation. Generally, the uptake of ions is more tightly regulated and occurs via different mechanisms compared to NPs.^53^ However, independently of uptake, extracellular ions may still elicit cellular effects. For example, nickel ions have been demonstrated to induce chromosomal damage by altering intracellular calcium and iron levels via extracellular interaction with cell-surface proteins/receptors.^54^ Together with the results of previous studies suggesting intracellular dissolution of AuNPs, released gold ions can act both intra- and extracellularly, and both potential dissolution processes need to be taken into consideration when studying the biosafety and fate of AuNPs.

## Conclusion

This study aimed to explore the macrophage-assisted dissolution of AuNPs of varying sizes and coatings, as well as underlying mechanisms. We have demonstrated that AuNPs dissolve to a larger extent in the presence of macrophages than cell medium alone, in a size- but not coating-dependent manner. Furthermore, the dissolution of 5 nm AuNPs was found to occur mainly extracellularly. Peroxynitrite, an oxidative species produced by macrophages, was shown to dissolve small AuNPs in cell medium and PBS, but not NaCl. Taken together, our results suggest extracellular dissolution of AuNPs in the presence of macrophages, likely due to the release of ROS/RNS in presence of ligands. These findings emphasize the importance of the presence of cells in the dissolution of NPs, as well as the fact that dissolution can occur extracellularly: two parameters that need to be taken into consideration in understanding the fate of AuNPs in the body.
